# More Than Just a Floppy Baby: Maintaining High Clinical Suspicion of Infant Botulism

**DOI:** 10.7759/cureus.102021

**Published:** 2026-01-21

**Authors:** Natalee Sarintra, Rachel Ekdahl, Sara C Sanders, Brittany Slagle, Katherine Tang

**Affiliations:** 1 Pediatrics, University of Arkansas for Medical Sciences, Little Rock, USA; 2 Pediatrics/Hospital Medicine, University of Arkansas for Medical Sciences, Little Rock, USA

**Keywords:** botulinum toxin, botulism, hypotonia, infant, lethargy

## Abstract

Infant botulism is a rare and life-threatening condition if left untreated. We report the case of a previously healthy four-week-old male in Arkansas, who presented with progressive lethargy, feeding difficulties, and respiratory compromise following a recent viral respiratory illness. His clinical course was notable for worsening hypotonia, absent reflexes, and eventual respiratory failure requiring intubation. Extensive evaluation for infectious, neurologic, and metabolic causes was initially unrevealing, but further exposure history revealed constipation paired with recent soil exposure, raising suspicion for infant botulism. Botulinum immune globulin (BabyBIG®) was administered, with subsequent gradual clinical improvement. Stool testing later confirmed the presence of botulinum toxin type B. The patient was discharged in stable condition, tolerating full oral feeds, and without further complications. This case underscores the diagnostic difficulty of infant botulism due to early age at presentation, anchoring bias with diagnosis of a recent viral illness, and overlapping symptoms with other neonatal conditions. A detailed exposure history is critical, and early clinical suspicion can facilitate timely treatment and improve outcomes.

## Introduction

Botulism is a rare but serious condition caused by colonization of the gastrointestinal tract by *Clostridium botulinum *spores, which produce botulinum neurotoxins that lead to neuromuscular blockade [1]. Infant botulism is the most common type of botulism and primarily affects infants under one year of age, with the highest incidence in those under six months [1].

Based on Centers for Disease Control and Prevention (CDC) data and data collected from the California Department of Health between 2007 and 2021, infant botulism demonstrated significant geographic variability, with disproportionately higher incidence reported in the United States (US) and Argentina compared with many other countries, possibly reflecting differences in Clostridium botulinum spore burden in soils and physician experience with the illness [2]. Within the US, the incidence of infant botulism per 100,000 live births was highest in the Northeast (4.9) and West (6.0) regions of the country, with a national average of 3.3 [2]. In Arkansas, the incidence during this period was 2.6 cases per 100,000 live births [2]. This regional variability may contribute to reduced clinical suspicion in lower-incidence states such as Arkansas.

Regardless of incidence, a thorough history and early recognition are critical, as disease progression can lead to neuromuscular weakness, respiratory failure, and death [3]. However, diagnosis of infant botulism can be challenging due to the subtle and nonspecific nature of early symptoms. For instance, affected infants commonly present with hypotonia, lethargy, poor feeding, a weak cry, and constipation [3,4]. These are features that significantly overlap with other neurologic, metabolic, or infectious diseases, which potentially introduce the risk of anchoring biases when considering workup for these patients.

We present a case of infant botulism in a previously healthy four-week-old male living in Arkansas. He was initially thought to have a viral respiratory illness, leading to a diagnostic delay, and highlighting the diagnostic complexity and the importance of thorough environmental exposure history in cases of unexplained neurologic decline in infants.

## Case presentation

A four-week-old male, born at full term by spontaneous vaginal delivery without complications, was transferred to our facility as a direct admission from an outside hospital due to concerns for feeding intolerance. He was admitted to the outside hospital for dehydration secondary to decreased oral intake in the setting of congestion that started two days prior. He otherwise did not have any fever, vomiting, or diarrhea. He tested positive for Rhinovirus/Enterovirus infection, to which his symptoms were attributed. He was admitted there for a total of two days, and throughout his stay, had difficulty latching to his bottle, warranting temporary nasogastric (NG) tube placement for feeding. He was noted to have intermittent choking episodes and progressive lethargy, for which he was transferred to our facility for further workup. Before transfer, he had a blood culture collected and was empirically started on ampicillin and ceftriaxone for antimicrobial coverage due to concerns for sepsis.

**Figure 1:**
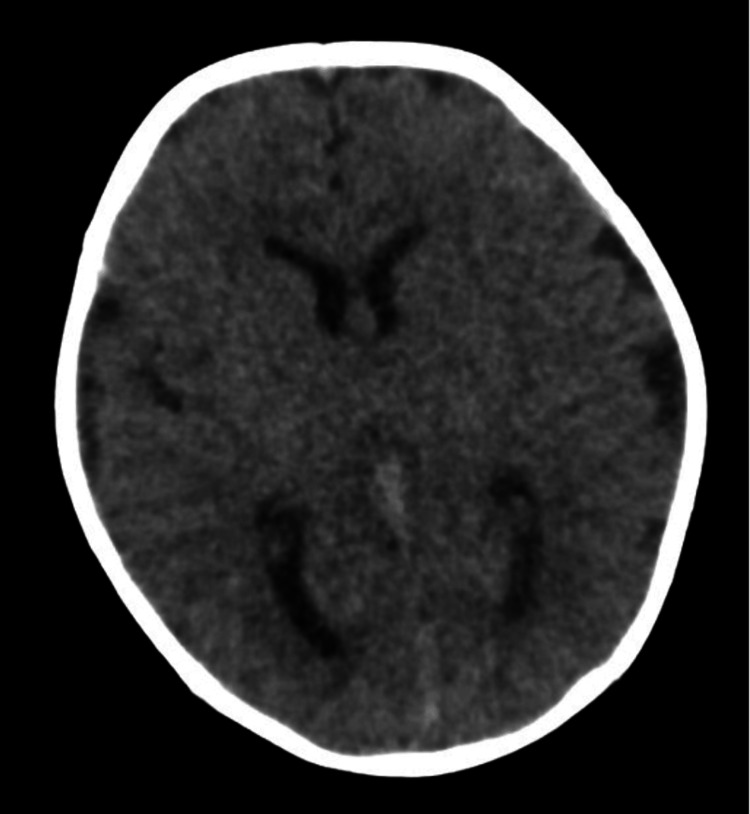
CT head without contrast showing no acute intracranial abnormalities. CT: computed tomography

He was transferred to our facility overnight (around 0200) on maintenance intravenous fluids. Following admission to our facility, a computed tomography (CT) head scan without contrast was obtained and was unremarkable, as shown below in Figure 1. A lumbar puncture was attempted but terminated due to desaturation following needle insertion; a second attempt done later in the afternoon (around 1200) was successful. Cerebrospinal fluid (CSF) studies were overall normal; cell count was without pleocytosis, gram stain and culture without growth, and the meningitis/encephalitis panel resulted in negative. Due to continued poor feeding, another NG tube was placed. However, on insertion (around 1300), the patient was noted to be significantly weaker, with a weak cry. He had shallow breathing and intermittent oxygen desaturations necessitating supplemental oxygen support with a nasal cannula. He was escalated to the pediatric intensive care unit (PICU). 

**Figure 2:**
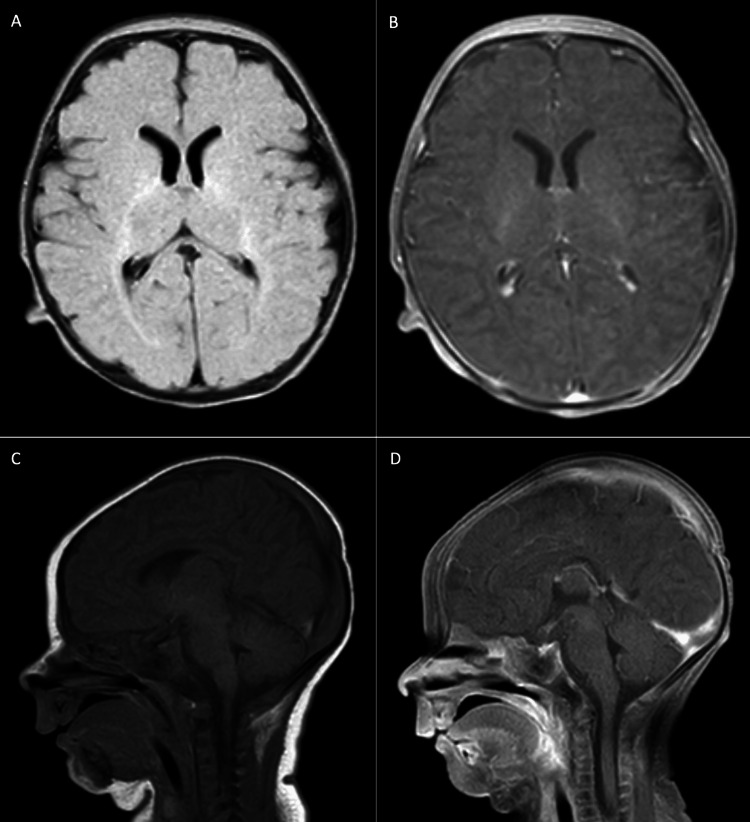
Normal MRI brain with and without contrast. A) Axial view of brain without contrast. B) Axial view of brain with contrast. C) Sagittal view of brain without contrast. D) Sagittal view of brain with contrast. MRI: magnetic resonance imaging

On examination in the PICU, he appeared diffusely hypotonic, with a weak cry and absent Moro, rooting, and grasp reflexes. With concerns for imminent respiratory failure, he was emergently paralyzed, sedated, and intubated (around 1600). Neurology, Genetics, and Infectious Diseases (ID) were consulted for additional recommendations. More workup for neurologic, metabolic, and infectious causes was performed. Magnetic resonance imaging (MRI) of the brain with and without contrast was normal, as shown below in Figure 2. MRI of the spine with and without contrast was also normal. He was monitored on video electroencephalogram (vEEG) without notable epileptiform activity. Urinalysis and urine drug screening were unremarkable. The neonatal newborn screen was confirmed to be normal. Serum ammonia, lactate, and pyruvate levels were normal. Plasma acylcarnitine profile, amino acids, and urine organic acids were unremarkable. Thyroid-stimulating hormone and free T4 levels were normal. Cytomegalovirus, Epstein-Barr virus, and Enterovirus blood testing were obtained and later resulted in negative results. 

On further history, parents reported he started with nasal congestion and decreased appetite about four days before this current admission, and his last bowel movement was about three days prior. Exposure history was significant for the patient being outside on a few instances while his father was fixing a fence, digging up, and moving dirt. Concerns for infant botulism increased due to the clinical presentation, including constipation, neurologic findings, and respiratory compromise. A stool sample was sent for botulinum toxin testing, and BabyBIG® (botulism immune globulin) was ordered from the California Department of Public Health on day 2 of admission. Antibiotics were discontinued due to concerns that bacterial lysis could increase the availability of botulinum toxin that could be absorbed, per Red Book Atlas of Pediatric Infectious Diseases guidelines [5]. BabyBIG® was administered on day three of admission.

The patient showed gradual clinical improvement. By day four, he was triggering spontaneous breaths, and by day five, he was intermittently more alert, with increased movement of the face and extremities. The patient was successfully extubated to a high-flow nasal cannula on day eight of admission. On day nine, he was transferred to the general pediatric floor. He was evaluated by a speech-language pathologist (SLP), who recommended continued NG tube feeds due to residual oral motor weakness and continued to assess him for oral intake readiness throughout the remainder of his admission. 

On day 10, he was weaned to room air. By day 11, SLP noted strong rooting and sucking on a gloved finger. A trial of oral feeding with thin formula and a level zero nipple was initiated. The patient was fed for 18 minutes but demonstrated fatigue and a weakening suck; the remainder of the feed was given via NG tube. On day 12, mildly thickened feeds were introduced every other feed to support better coordination and endurance, and on day 13, the patient was cleared for thin liquids. Over the next several days, he worked with SLP to increase oral intake, with the goal of achieving all oral feedings. By day 17, the patient was tolerating full oral feeds, and the NG tube was removed. He was discharged home in stable condition. 

On day 18, the results of the stool test returned positive for botulinum toxin type B. ID followed up with the family, who reported that the infant was doing well at home with no concerns. 

## Discussion

Maintaining a high index of suspicion is essential to the diagnosis and management of infant botulism. The nonspecific nature of early manifestations, such as poor feeding, lethargy, and hypotonia, overlaps with a broad spectrum of neonatal and infant disorders, thereby complicating timely recognition. Additionally, the median age of presentation is approximately 10 weeks [5], making diagnosis in younger infants, as in this case, more challenging. In regions where infant botulism incidence is lower than the national average, clinicians may be less likely to suspect botulism, making history-taking especially critical to direct diagnostic reasoning. 

Assessment of potential exposure sources represents a key component of the history. While ingestion of honey or other solids containing Clostridium botulinum spores is a well-established risk factor for infant botulism, environmental exposure to soil or dust containing spores is also recognized. After ingestion, spores travel to the intestine, where they germinate, multiply, and produce botulinum toxin [6]. The most common toxin types causing infant botulism are A and B [6], with type B identified as the culprit in this case. The patient had no known ingestion of contaminated food, but was outdoors while his father was working with disturbed soil. The distance between the patient and the disturbed soil, duration of exposure, and whether airborne dust was visibly present are not known, presenting a limitation to this case report; however, this exposure was considered a likely opportunity for environmental contact with Clostridium botulinum, as there were no other known exposures.

The diagnostic process was further complicated by the initial attribution of symptoms to a documented Rhinovirus/Enterovirus infection. This early interpretation introduced the potential of anchoring bias, in which clinicians may focus on an early or more apparent diagnosis, leading to delayed recognition of other alternative etiologies. Getting more history from the patient's family led to recognition of an environmental exposure and constipation. This case emphasizes the importance of continually revisiting and broadening the differential diagnosis throughout a patient’s hospital stay, especially when the clinical course deviates from expectations or fails to improve as anticipated. 

A wide range of conditions can mimic the presentation of infant botulism, often prompting more extensive workups. These include central nervous system infections, traumatic injuries, neurodegenerative processes, seizure disorders, or conditions causing metabolic derangements [7-12]. Some of the differential diagnoses considered in this patient's case are listed in Table 1.

**Table 1:** Common mimics of infant botulism References: [7-10]

Diagnosis	Typical presenting symptoms
Head trauma	Fussiness, poor feeding, vomiting, lethargy
Meningitis/encephalitis	Fever versus hypothermia, poor feeding, vomiting, rash, lethargy
Guillain-Barré syndrome	Ascending paralysis often following viral or bacterial illness, areflexia, hypotonia, weakness, and constipation^7^
Spinal muscular atrophy	Absent deep tendon reflexes, sparing of extraocular muscle paralysis, history of several weeks of progressive weakness^8^
Myasthenia gravis	Poor feeding, weak suck, weak cry, hypotonia, facial diplegia^9^
Acute flaccid myelitis	Acute onset of focal or asymmetric weakness, poor feeding, dysphagia, respiratory distress^10^
Epilepsy	Seizure activity, impaired level of consciousness
Inborn error of metabolism	Poor feeding, poor weight gain, electrolyte abnormalities, hypoglycemia
Congenital hypothyroidism	Poor feeding, lethargy, hypotonia

Recognition of these potential mimics underscores the need for vigilance and systematic evaluation. In this case, the patient had negative CSF studies, ruling out infectious processes like meningitis, overall normal metabolic labs, ruling out inborn errors of metabolism and congenital hypothyroidism, normal imaging and vEEG findings, ruling out head trauma, inflammatory spinal infections, and epilepsy. Paired with a history of normal development, organic neurologic etiology was felt to be less likely. Moreover, the rapid deterioration over the course of days made the consideration of infant botulism higher and spinal muscular atrophy (SMA) versus other muscular dystrophies less likely.

Early diagnosis of infant botulism is crucial. As the neurotoxin impairs neuromuscular transmission, untreated progression can result in neurologic deficits, such as cranial nerve palsies, and respiratory muscle paralysis [3, 7]. In this case, the patient required intubation and mechanical ventilation due to rapidly worsening hypotonia and respiratory status. Prompt clinical suspicion, including obtaining a detailed history that may include poor feeding, weakness, and constipation, along with recognition of impending respiratory failure, and contacting the California Department of Public Health Infant Botulism Treatment and Prevention Program to help provide the botulism immune globulin (BabyBIG®) was done, and administration of the medication was instrumental in halting disease progression and facilitating recovery. 

Ultimately, this case demonstrates the importance of maintaining a broad differential diagnosis in the evaluation of a hypotonic, lethargic infant and highlights the critical role of environmental history in identifying cases of infant botulism. Early recognition and intervention can significantly alter the clinical course and improve outcomes in affected infants. 

## Conclusions

Infant botulism should remain a critical consideration in the differential diagnosis of infant weakness, particularly in the presence of progressive hypotonia, respiratory distress, constipation, and feeding difficulties. Avoiding anchoring bias and obtaining a thorough history are essential to establishing the diagnosis, especially when clinical deterioration is incongruent with initially unrevealing laboratory and imaging evaluations. This case illustrates the diagnostic complexity of infant botulism and highlights the decisive role of a detailed environmental exposure and feeding history, as well as the necessity of early clinical suspicion, to enable timely treatment and improve patient outcomes.
